# How Cells Can Control Their Size by Pumping Ions

**DOI:** 10.3389/fcell.2017.00041

**Published:** 2017-05-08

**Authors:** Alan R. Kay

**Affiliations:** Department of Biology, University of IowaIowa City, IA, USA

**Keywords:** osmosis, ion transport, sodium chloride, potassium, impermeant anions, Donnan effect, Na^+^/K^+^ ATPase

## Abstract

The ability of all cells to set and regulate their size is a fundamental aspect of cellular physiology. It has been known for sometime but not widely so, that size stability in animal cells is dependent upon the operation of the sodium pump, through the so-called pump-leak mechanism (Tosteson and Hoffman, 1960). Impermeant molecules in cells establish an unstable osmotic condition, the Donnan effect, which is counteracted by the operation of the sodium pump, creating an asymmetry in the distribution of Na^+^ and K^+^ staving off water inundation. In this paper, which is in part a tutorial, I show how to model quantitatively the ion and water fluxes in a cell that determine the cell volume and membrane potential. The movement of water and ions is constrained by both osmotic and charge balance, and is driven by ion and voltage gradients and active ion transport. Transforming these constraints and forces into a set of coupled differential equations allows us to model how the ion distributions, volume and voltage change with time. I introduce an analytical solution to these equations that clarifies the influence of ion conductances, pump rates and water permeability in this multidimensional system. I show that the number of impermeant ions (*x*) and their average charge have a powerful influence on the distribution of ions and voltage in a cell. Moreover, I demonstrate that in a cell where the operation of active ion transport eliminates an osmotic gradient, the size of the cell is directly proportional to *x*. In addition, I use graphics to reveal how the physico-chemical constraints and chemical forces interact with one another in apportioning ions inside the cell. The form of model used here is applicable to all membrane systems, including mitochondria and bacteria, and I show how pumps other than the sodium pump can be used to stabilize cells. Cell biologists may think of electrophysiology as the exclusive domain of neuroscience, however the electrical effects of ion fluxes need to become an intimate part of cell biology if we are to understand a fundamental process like cell size regulation.

## Introduction

Cells in organisms of all phyla are typically small, with diameters of a few tens of microns. This length scale allows for the rapid diffusion of molecules in the cytoplasm (Berg, 1993). If the cells were larger by an order of magnitude the average diffusion time across a cell would rise by two orders of magnitude. It is this rapid growth of transit times that in part sets a very tight limit on absolute cell size.

There is abundant evidence that cells are able to control their size (Marshall et al., 2012; Amodeo and Skotheim, 2016), but little as to how they do so. Here I will show that there is a tight link between cell size, membrane potential and impermeant intracellular molecules, at least for cells with pliant membranes. At first glance in the huge array of cellular factors, these may seem somewhat remote but they are tied together through the osmotic movement of water across the plasma membrane. This connection can be all but invisible, unless one exposes the forces that drive ion and water fluxes.

The monovalent inorganic ions, Na^+^, K^+^, and Cl^−^ are, next to water, the second most abundant components of cells (Frausto da Silva and Williams, 2001). These ions play central roles in the energetics of cells and in determining the osmotic stability of cells. In most cell biology textbooks they are often given short shrift, relegated to counter-ions that play a bystander role. There is perhaps a tendency in cellular biology to locate the drivers of cellular activity in the interactions between macromolecules. The province of ions and potentials is often only seen as germane in neurophysiology; however, I will argue that it is a powerful determinant of cell biology.

There are conceptually two forms of cell size regulation that can be distinguished. First, the processes that determine the size distributions of various cell types in an animal that I will term cell size regulation (CSR), which for example, makes fibroblasts larger than hepatocytes (Ginzberg et al., 2015). Second, there are mechanisms that stabilize the cell volume when the osmolarity of the extracellular fluid changes, which I will call cell volume regulation (CVR, reviewed in Hoffmann et al., 2009). Although there are likely to be links between these two processes, I will focus on CSR in this paper.

All cells have a problem that stems from their need for an inventory of impermeant molecules (metabolites, proteins, nucleic acids, etc., Burton, 1983b) that sets up an unstable osmotic condition, which could rupture the plasma membrane if left unattended (Stein, 2002; Armstrong, 2003; Dawson and Liu, 2008). This is the so-called Donnan effect (Sperelakis, 2012). Plants and prokaryotes solve the problem by building cell walls that can resist turgor pressure (Haswell and Verslues, 2015; Wood, 2015). Some single cell eukaryotes pump out excess water (Allen and Naitoh, 2002). All animal cells appear to remedy the problem by pumping Na^+^ out and K^+^ in with a Na^+^/K^+^ ATPase (NKA), while allowing the passive leak of ions and water down their gradients (Weiss, 1996). The Na^+^ and K^+^ gradients also serve as energy reservoirs for transporting other molecules against their concentration gradients and in establishing a negative resting membrane potential.

Tosteson and Hoffman (1960) demonstrated how the operation of the NKA can stave off an osmotic catastrophe, where water flows into the cell until it lyses. Although their so-called pump-leak model (PLM) is well established and part of the standard canon of physiology (Boron and Boulpaep, 2016), precisely how it works has not been widely disseminated. I will argue that this is so because, for the most part, the understanding of electrical current flow is not considered a necessary part of a cell biologist's intellectual toolbox. Part of my aim is to show how this omission can hobble our comprehension of a crucial aspect of cell biology and to remedy it by providing the essentials of what one needs to know, to understand the fundamentals of ion and water flow.

Post and Jolly (1957) were the first to show theoretically, how pumping a permeant molecule could stabilize a cell containing impermeant molecules, however their model only considered uncharged molecules. In 1960, Tosteson and Hoffman demonstrated that erythrocyte volume was stabilized by the action of a NKA, which in essence prevents the influx of water induced by the presence of impermeant molecules in the cell. They showed that it was not the NKA alone that was responsible for volume stabilization but a coupled system, which includes Na^+^, K^+^, Cl^−^, and water permeability. The action of the NKA can be viewed as making room for the impermeant ions in the cell and equalizing the osmotic pressure across the membrane. The PLM accounts for the asymmetric distribution of Na^+^ and K^+^ across animal cells first observed by Schmidt (1850) and Clarke and Fan (2011). This distribution of ions is not just a peculiarity of animal cells but is a universal characteristic of cells in all phyla (Somero et al., 2017).

It is not my purpose in this paper to examine the experimental evidence for the PLM (Macknight and Leaf, 1977; Hoffmann et al., 2009) in stabilizing cell size but to assess and clarify aspects of its theoretical foundations. I believe that the PLM cannot be understood fully without tackling its mathematics. Here I show how one can set up a system of equations, derived from physical laws (Sterrat et al., 2011; Nelson, 2014), which model the interaction between ion and water fluxes, allowing one to calculate both the membrane potential (*V*) and cell volume (*w*). In addition, I introduce an analytical solution derived by Keener and Sneyd (2009), which is useful in making clear how the ionic conductances, pump rate and ion concentrations influence *w* and *V*. Using these equations I show that there is direct connection between the number of impermeant molecules (*x*) in a cell and its volume.

Electrophysiology is often considered to be the province of neuroscience. In this paper I hope to show that it is an essential tool for understanding cells and how they regulate their size.

## Methods

All simulations and calculations were performed in MATLAB (Mathworks, Natick, MA) and graphs were plotted using Origin (MicroCal, Northampton, MA). Numerical integration of the differential equation was by the simple Euler method with step size 1–100 μs (See Supplementary Material for MATLAB program).

### Symbols and abbreviations

*A*, Membrane area
*C*, Membrane capacitance
*F*, Faraday's constant
g*_i_*, Conductance of ion *i*
*n*, Number of Na^+^ pumped per NKA cycle
*p*, Pump rate
*P*_*f*_, Osmotic water permeability coefficient
*q*, Number of K^+^ ions pumped per NKA cycle
*R*, Universal gas constant
*T*, Absolute temperature
*w*, Cell volume
*x*, Number of moles of the impermeant intracellular molecules
*z*, Average charge of impermeant intracellular molecules
*V*, Voltage
Π, The intracellular osmolarity
Π_*o*_, The extracellular osmolarity.

### Default values of parameters

Note that this parameter set has been used in all figures unless otherwise specified.

Cl_*o*_ = 150 mM
C_*m*_ = unit membrane capacitance = 1 μF cm^−2^
g_*Cl*_ = 0.2 mS cm^−2^
g_*K*_ = 0.3 mS cm^−2^
g_*Na*_ = 0.01 mS cm^−2^
K_*o*_ = 3 mM
*n* = 3
Na_*o*_ = 147 mM
*q* = 2
*T* = 25°C
*x* = 2.6 × 10^−14^ mole
z = −1

### The pump-leak mechanism

The work presented here derives from a long line of work in cellular physiology. I thought it useful to present it in part as a tutorial, since some of the information is collected in rather specialized publications, often directed at excitable cells. I wanted to present it as one concise narrative rather than as a tedious opera. I hope to make clear that there is a direct connection between the number of impermeant ions in a cell and its size. The elementary physics that is required to understand electrical current flow in biological systems is succinctly covered in the appendices of the following textbooks (Nicholls et al., 2011; Blaustein et al., 2012).

In what follows I will set up a model that incorporates all the forces and constraints that act on ions and water, both within and outside the cell, to determine how the ions and water will distribute as time moves on. A crucial feature of the model is that the membrane is assumed to be freely distensible, so that as water moves in it will expand correspondingly and shrink if water moves out.

In addition, I will assume that the composition of the solution in the extracellular space is fixed, which is reasonable since the volume of the extracellular space if far larger than that of a single cell. I will also set aside spatial effects and assume that the cell, for the purposes of this paper, can be considered a single iso-potential sphere (i.e., all points within the cell are at the same potential) and the voltage in the extracellular space is zero.

To improve the flow of the equations I have adopted the following conventions: capitalized solutes represent concentrations, and lower cases represent amounts in moles. Extracellular ions are denoted by a subscript “o” for “outside,” intracellular ions have no subscript.

### The Donnan effect

Animal cell membranes, being somewhat fragile, cannot sustain much transmembrane pressure nor expand much (see however Sachs and Sivaselvan, 2015). Therefore, because membranes are permeable to water, the osmolarity of the extracellular fluid should match closely that of the intracellular fluid. Over 70 years ago Boyle and Conway (1941) realized that the impermeant molecules (metabolites, proteins, nucleic acids etc.) which cells must contain, impose an osmotic load on cells.

To see how this arises I will consider the effect of impermeant ions on the osmotic balance of cells. Since the osmotic strength of a solution is a colligative property (Atkins and de Paula, 2014), all the impermeant molecules can be lumped together and considered as a single chemical species, without any loss of precision, which I will term *x* (where *X* is its osmolarity and *x* the number of osmoles) with an average charge *z*. It should be noted that some molecules might have osmotic coefficients less than one.

It can be shown (see below) that a cell which has a pliant membrane, permeant to monovalent ions and containing impermeant molecules, immersed in a saline solution cannot reach a stable steady state without the input of energy and will burst (Weiss, 1996). Whereas in the absence of impermeant molecules the “cell” is stable if the solution within the cell is identical to that outside.

The influence of the impermeant molecules is called the Donnan (or Gibbs-Donnan) effect, which has been and continues to be a concept that has been misapplied and misunderstood even in contemporary textbooks. Although a Donnan equilibrium is not possible in a live animal cell, *x* does exert an effect on the cell and cannot be ignored. Tosteson and Hoffman (1960) showed that the operation of an energy consuming NKA stabilizes cell volume in the presence of *x* and water permeability. It is this PLM that I will discuss in the rest of this article.

### The constraint equation

To see how *x* and *z*, together with the distributions of Na^+^, K^+^, and Cl^−^ determine cell volume I consider two physico-chemical constraints on cells:

1. **Osmotic balance**. Since the membrane of animal cells cannot support much of a transmembrane pressure, the osmotic strength of the intracellular solution should be equal to that of the extracellular solution (Π_o_):
  (1) $$\begin{array}{l} Na\;+\;K\;+\;Cl\;+\;X\;=\;\Pi_{o} \end{array}$$
2. **Charge Balance**. The number of positive charges inside the cell should match that of the intracellular negative charges
  (2) $$\begin{array}{l} Na\;+\;K\;-\;Cl\;+\;z\frac{x}{w}=0 \end{array}$$

Where *w* is the cell volume. This also holds true for the extracellular solution.

From these two conditions I can derive an expression for the cell volume:

(3) $$\begin{array}{l} w=\frac{\left(1-z\right)x}{\Pi_{o}-2Cl}=\frac{\left(1-z\right)x}{\Pi_{o}-\;2Cl_{o}e^{FV/RT}} \end{array}$$

Where *V* is the transmembrane potential. The charge imbalance that gives rise to the transmembrane potential is so small as to be chemically undetectable (Burton, 1975). The only assumption for the second part of this equation is that the chloride distribution is at equilibrium.

From this equation it is clear that the volume has a simple dependence on *x* and its charge, *z*. This equation was derived by Boyle and Conway (1941), and I follow Fraser and Huang (2004) in calling it the “constraint equation.”

**Figure 1:**
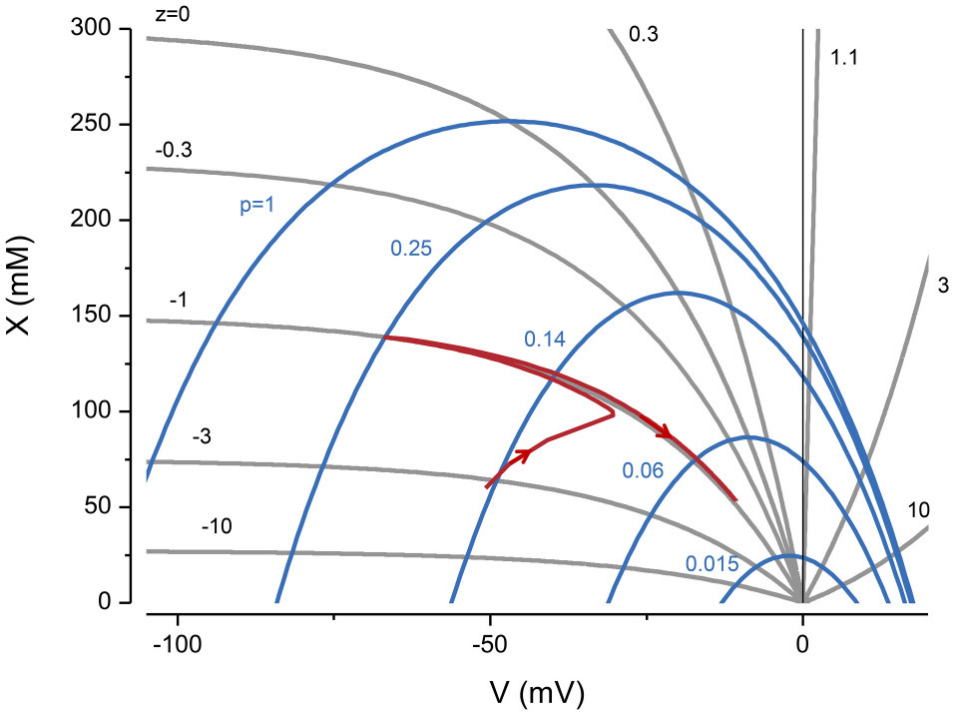
**The influence of ***z*** and pump rate (***p***), through the constraint equation, on ***V*** and ***X*****. The gray lines are from Equation (3) and the blue ones from the KSSs. *p* in (C mm^−2^ s^−1^). The red line represents the trajectory of a system with *z* = −1 and *p* = 0.25 C mm^−2^ s^−1^, where the pump is turned on and then off after reaching a steady state.

The constraint equation shows for a given *z*, which values of *X* and *V* are consistent with a stable volume (Figure 1, see gray lines). However, it only determines the sum of Na^+^ and K^+^. To determine the individual concentrations, I have to consider the forces acting on all the ions.

Two important implications of the constraint equation are that to accommodate *x*, the voltage needs to be negative, and with a voltage of zero the volume goes to infinity i.e., the cell is unstable. A useful way of thinking about the PLM is that it creates a negative potential and Cl^−^ follows its equilibrium making space to accommodate *x*.

### The ion flux equations

To complete the model of the cell, equations are required that describe the forces acting on the three mobile ions, Na^+^, K^+^, and Cl^−^. These forces are chemical potential (diffusion), electric, and active ion transport. This gives three equations for the rate of change of the intracellular ion concentrations:

(4) $$\begin{array}{l} \frac{dNa}{dt}=-\frac{A}{wF}\left({\text{g}}_{Na}\left[V-\frac{RT}{F}ln\left(\frac{\text{N}{\text{a}}_{o}}{Na}\right)\right]+np\right)-\frac{1}{w}\frac{dw}{dt}Na \end{array}$$

(5) $$\begin{array}{l} \frac{dK}{dt}=-\frac{A}{wF}\left({\text{g}}_{K}\left[V-\frac{RT}{F}ln\left(\frac{{\text{K}}_{o}}{K}\right)\right]-qp\right)-\frac{1}{w}\frac{dw}{dt}K \end{array}$$

(6) $$\begin{array}{l} \frac{dCl}{dt}=\frac{A}{wF}\left({\text{g}}_{Cl}\left[V+\frac{RT}{F}ln\left(\frac{\text{C}{\text{l}}_{o}}{Cl}\right)\right]\right)-\frac{1}{w}\frac{dw}{dt}Cl \end{array}$$

**Figure A1:**
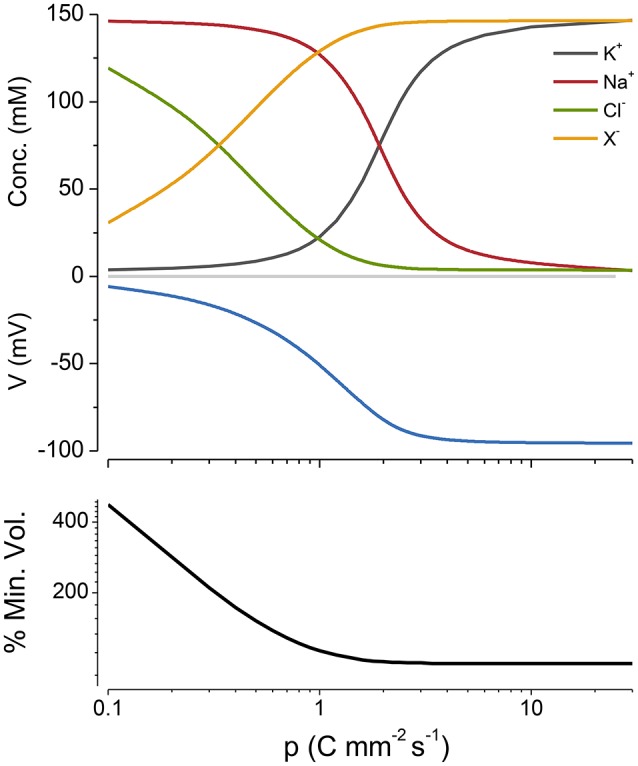
*****Cp*** plot of PLM with a nonlinear NKA mechanism (Equations A13 and A14)**. *D*_*Na*_ = 0.8 mM and *D*_*K*_ = 3.6 mM. The % of the minimum volume is plotted as function of *p* on the lowest panel.

Where *A* is the membrane area (assumed to be constant) and *F* Faraday's constant. To break the expressions down into their components, the term ${\text{g}}_{Na}\left[V-\frac{RT}{F}ln\left(\frac{\text{N}{\text{a}}_{o}}{Na}\right)\right]$ is Ohm's law, which combines the chemical and electrical potentials, and $\frac{RT}{T}ln\left(\frac{Na_{o}}{Na}\right)$ is often referred to as the “Nernst potential.” While, the NKA is represented by a constant pump rate *p* with *n* and *q* being the number of Na^+^ and K^+^ ions, respectively transported per cycle. As I show in the **Appendix** (**A2**, Figure A1) the simplified form of the NKA, where it is represented by constant terms in Equations (4) and (5), has little impact on my overall conclusions. The last term in the equations reflects the influence of volume changes on ion concentrations. This term is very small and can be dropped without incurring much error.

The assumption of constant area is adopted to reflect the fact that over a short time scale the number of channels and transporters is unlikely to change much.

The flow of water can be modeled by the following equation (Fettiplace and Haydon, 1980):

(7) $$\begin{array}{l} \frac{dw}{dt}=\nu_{w}P_{f}\text{A}\left(\Pi -\Pi_{o}\right) \end{array}$$

Where *P*_*f*_ is the osmotic water permeability coefficient of the membrane and ν_w_ is the partial molar volume of water (18 cm^3^ mol^−1^). Water permeability even in the absence of aquaporins is higher than the ionic permeability (Verkman, 1992) and I will assume that water instantaneously equilibrates across the membrane, unless otherwise noted.

From the definition of capacitance, the voltage is given by the following equation (Varghese and Sell, 1997):

(8) $$\begin{array}{l} \text{V}=\frac{Q}{C}=\frac{Fw}{AC_{m}}(Na+K-Cl+zX) \end{array}$$

I shall refer to the system of Equations (1), (2), (4), (5), (6), and (8) as the “pump-leak equations” (PLE). In 1998, Keener and Sneyd found an analytical solution for the steady state of the PLEs, but this important work has as yet not made its way into the biological literature. The advantage of an analytical solution is that it makes evident the influence of the various factors at play in regulating cell volume. Moreover, it offers a convenient check of numerical methods used for solving the PLEs. Keener and Sneyd's solutions (KSS) to the PLEs are given in **Appendix A1**.

### Integrating the PLEs

No closed-form solutions are available for the approach of the system to steady-state, hence the system has to be solved by numerical integration.

To solve numerically the PLEs I integrate Equations (4–7) and use the algebraic Equation (8) to calculate *V*. Fraser and Huang (2007), termed this the “charge-difference” (CD) method, which explicitly takes into account the impermeant ions, *x* and their charge, *z*. I have found that the CD approach converges to the KSS, with reasonable initial conditions (data not shown).

**Figure 2:**
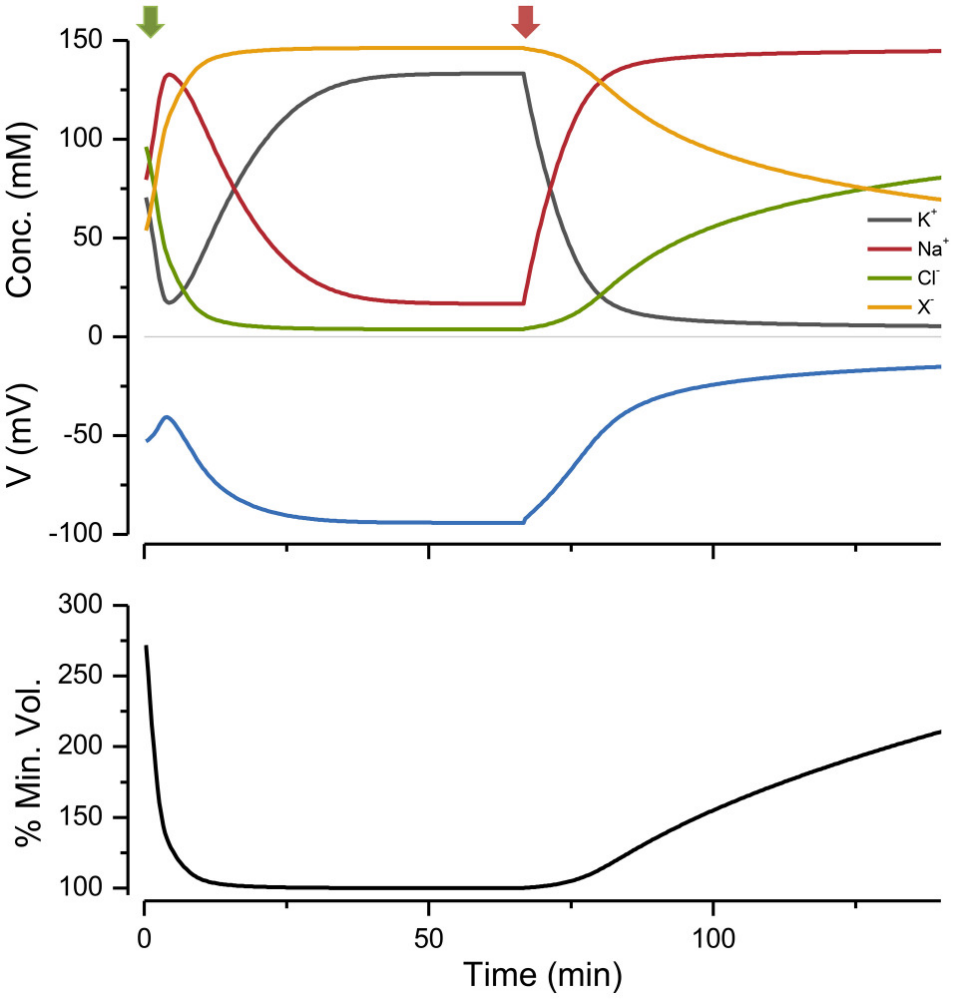
**The effect of turning the NKA on and then off, on ion concentrations, voltage and size**. Green arrow, pump on; red, pump off. *p* = 0.5 C mm^−2^ s^−1^. The % of the minimum volume is plotted as function of time on the lowest panel.

**Figure 3:**
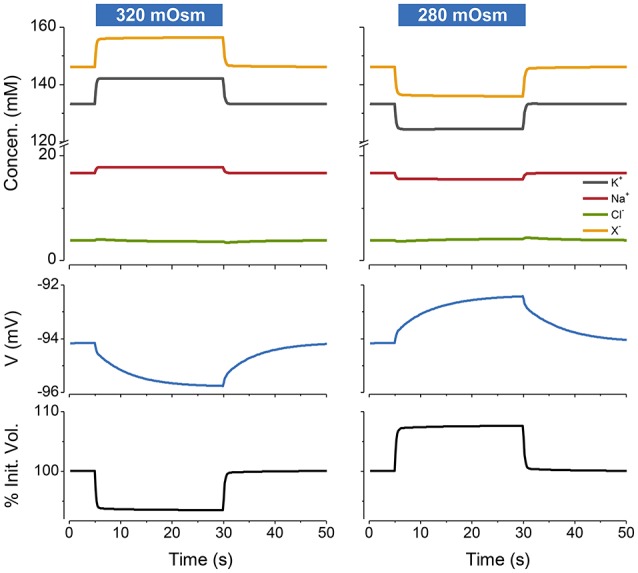
**The PLM stabilizes cells against changes in extracellular osmolarity**. The blue rectangles over the figures indicate the period during which the osmolarity was changed from the control value of 300–320 mOsm (left panel) or 280 mOsm (right panel). Water is removed or added respectively to the default extracellular solution. *p* = 0.5 C mm^−2^ s^−1^. The osmotic water permeability = 0.05 cm s^−1^. The % of the initial volume is plotted as function of time on the lowest panel.

If I begin the simulation with some arbitrary values of ion concentrations and turn on the NKA the system approaches the steady state given by the KSS. If after reaching steady state the NKA is turned off, the ions equilibrate across the membrane and the volume increases continuously, showing that the system is unstable in the absence of NKA activity (Figure 2). An important feature of the PLM is its robustness; a stable volume can be achieved with a very wide array of combinations of channel conductances and extracellular ion concentrations, so long as $n\frac{Na_{o}}{g_{Na}}>q\frac{K_{o}}{g_{K}}$ and the *p* not too high (Keener and Sneyd, 2009; Mori, 2012). The PLM also stabilizes cells against sudden changes in extracellular osmolarity, which is followed by the cell changing to a new stable steady state volume (Figure 3).

It is worth noting that if a cell has no impermeant ions, which is clearly impossible, the system is stable in the absence of an NKA. However, with no *x*, the operation of an NKA has the paradoxical effect of rendering the system unstable (see **Appendix A3**).

### The effect of *z*

**Figure 4:**
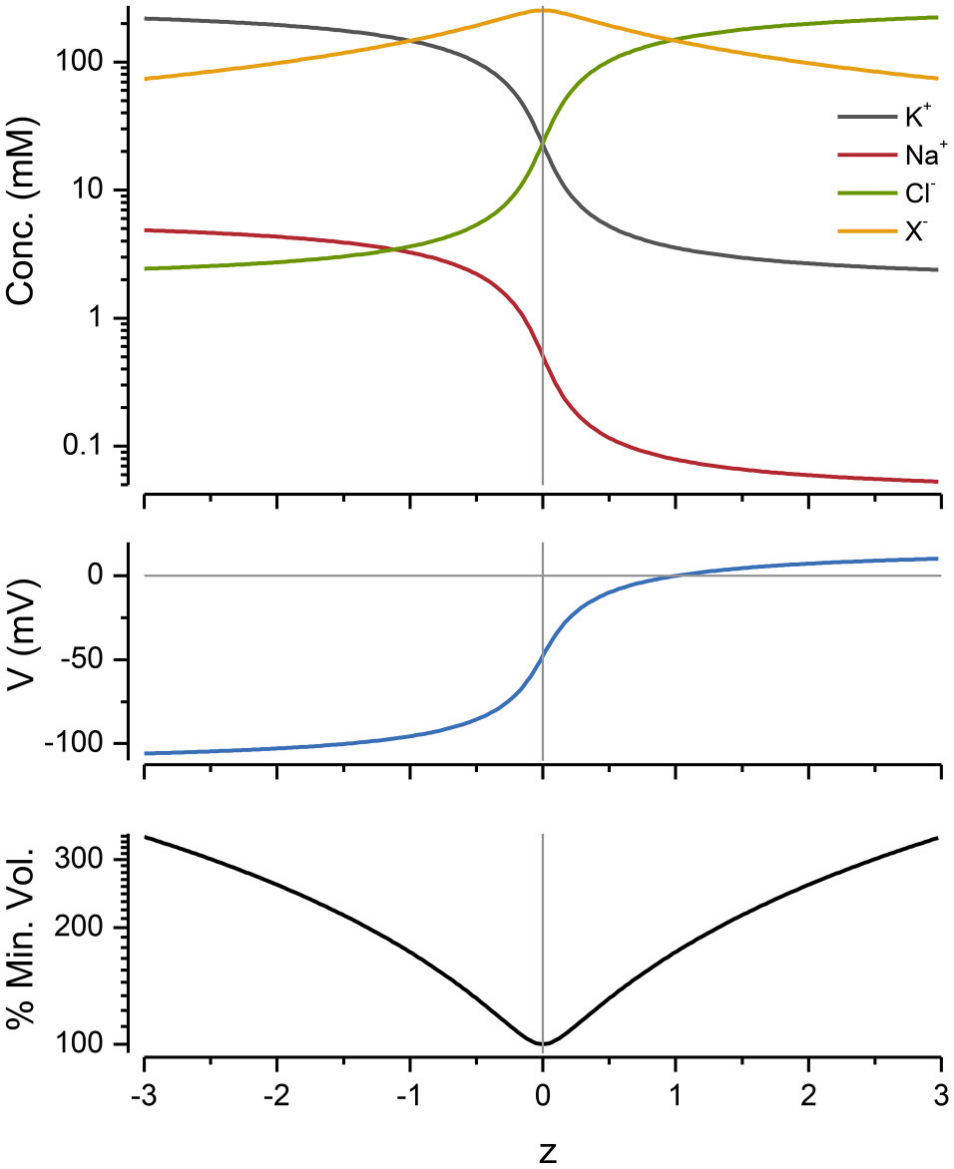
**The effect of ***z*** on ion concentrations, voltage and cell radius**. *p* = *p*_*min*_ and was 0.65 C mm^−2^ s^−1^ for all of the values shown. The % of the minimum volume is plotted as function of *z* on the lowest panel.

The impermeant molecules *x* exert an osmotic effect on the system and constrain the size of the cell. The mean charge on these molecules, *z*, also has a powerful influence on the ion distributions, voltage and volume of the cell. This can be seen by plotting these variables as a function of *z* with a fixed number of moles of *x* (Figure 4).

### *Cp* curves

**Figure 5:**
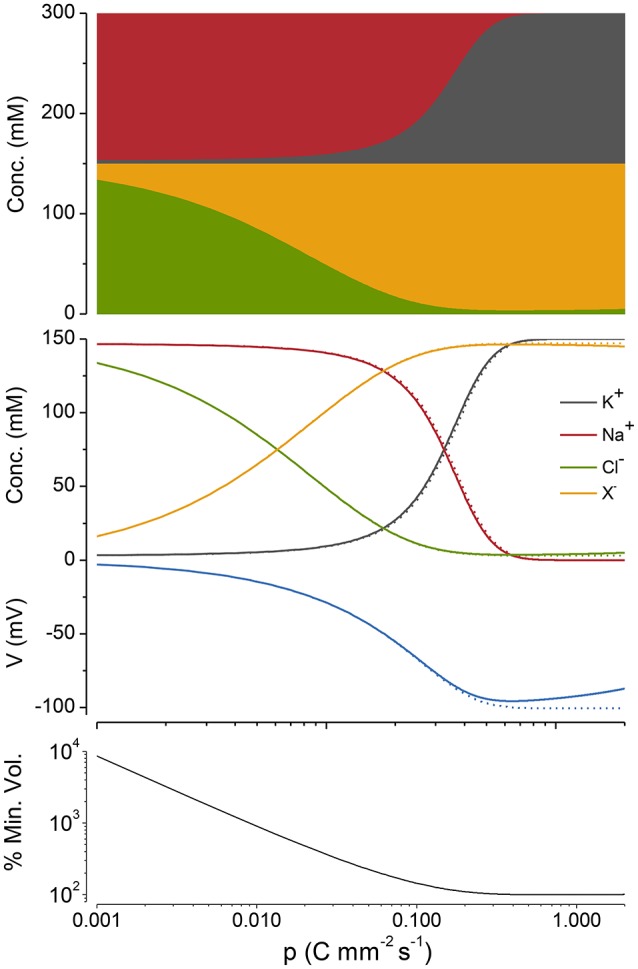
**A ***Cp*** plot of ion concentrations, voltage and cell radius**. *z* = −1, *n* = 3, and *q* = 2. The case where potassium in not actively transported (*q* = 0) is shown in gray. The % of the minimum volume is plotted as function of *p* on the lowest panel.

The action of the NKA can be made evident by plotting ion concentrations, *V* and volume as a function of the pump rate, *p* What I will term, *Cp* curves, provide a way of visualizing the forces at play as *p* is ramped up and the effect of *p* on the apportioning of ions. When one plots the concentration of intracellular ions as a function of pump rate the ion concentrations form a braid (symmetrical for the case of *z* = −1 because of the osmotic and charge constraints (Figure 5, 2nd panel from the top and see **Appendix A4**).

**Figure A2:**
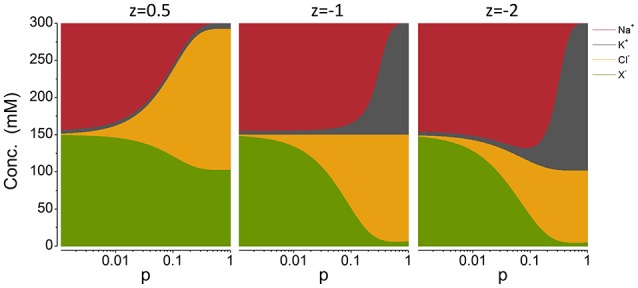
*****Cp*** plots with different ***z***′***s***, noted on the top of each panel**. The pump rate *p* is in C mm^−2^ s^−1^.

If, as in Figure 5 (top panel), the cations and anions in the *Cp* plots are grouped separately, the preservation of charge and osmotic balance become obvious; the sum of all intracellular species is equal to Π_*o*_, while the sum of the positive charges is equal to that of the negative charges. This also holds for *z* ≠ −1 but relationships between the ion concentrations and *p* become somewhat more complicated (see **Appendix A5**, Figure A2).

As *p* is increased, although Na^+^ and K^+^ are being pumped, it is Cl^−^ and *X* that first respond to this applied force. Why is this so? Because the net action of the NKA during a cycle is to move one positive charge out of the cell, it makes the inside of the cell more negative (i.e., hyperpolarizes), which drives Cl^−^ out of the cell passively requiring little energy, while setting up the asymmetric Na^+^/K^+^ distribution, which is far out of equilibrium, requires a greater energetic input.

An alternate way of viewing the PLM is shown in Figure 1 where *X* vs. *V* derived from the KSEs is plotted for a given *p* (blue lines). The intersections between the CE equation and the *X* vs. *V* serve as attractor points, so that if the system starts at a nonequilibrium point it will move to the intersection when the pump is turned on. If the pump is turned off the system moves to *V* = 0 and the volume goes to infinity.

### Mathematical knockout of components of the NKA

The PLEs can be further simplified by assuming that the NKA can actively transport only one of the monovalent cations.

If Na^+^ transport is eliminated but all other aspects of the PLM are left intact, no stable solutions are possible (see **Appendix A6**). This happens because the pump generates an inward current that depolarizes the cell and draws Cl^−^ in. Na^+^ cannot follow the voltage because of its high extracellular concentration and it remains out of register with the *V* throughout the *Cp* curve. This mechanism is incapable of generating a stable volume and generates a strong depolarization.

If K^+^ transport is eliminated but all other aspects of the PLM are left in place, there is very little difference in the operation of the mechanism as seen in a *Cp* plot (Figure 5). K^+^ and Cl^−^ can move passively and attempt to follow the voltage. As mentioned above the Na^+^ pump hyperpolarizes the cell. This drives Cl^−^ out and K^+^ in. For this case, the K^+^ and Cl^−^ follow the Donnan relationship (viz. *K Cl* = *K*_*o*_*Cl*_*o*_) exactly and are both in equilibrium with the voltage (see **Appendix A6**). Because the system requires ATP to sustain a steady-state, the system as whole is not at equilibrium.

The actual NKA requires both Na^+^ and K^+^ to operate, however my analysis shows that Na^+^ transport is the only necessary component if one thinks of its role in volume regulation. Dropping active K^+^ transport simplifies the PLEs so that it becomes possible to arrive at analytical solutions to the intersection points on *Cp* plots and to see how they depend on the systems parameters (see **Appendix A4**).

### The effect of Cl^−^ conductances

The effect of the Cl^−^ conductance on the PLM is entirely passive but it is an essential one, since if the conductance is blocked Cl^−^ cannot equilibrate and the mechanism is blocked. In many cells Cl^−^ is not passively distributed but is pumped and this will have an effect on the volume regulatory mechanism but is beyond the scope of this paper (Kaila et al., 2014; Vereninov et al., 2016).

### Optimizing the pump rate

The pump rate (*p*) is a direct measure of the energy needed to sustain the volume and voltage of the cell, since it is directly coupled to ATP hydrolysis. In the PLM, as the pump rate is increased, the volume declines and reaches a minimum at what I will call p_*min*_ (Figure 5). If *p* is increased above this, the volume then increases very slowly. For the case where *z* = −1, p_*min*_ can be found by differentiating Equation (A12) with respect to *p*, setting this derivative equal to zero and solving for *p*:

(9) $$\begin{array}{l} {\text{p}}_{min}=\frac{F}{RT}\left(\frac{qg_{Na}+ng_{K}}{g_{K}g_{Na}}\right)ln\left(\frac{ng_{K}Na_{o}}{qg_{Na}K_{o}}\right) \end{array}$$

**Figure 6:**
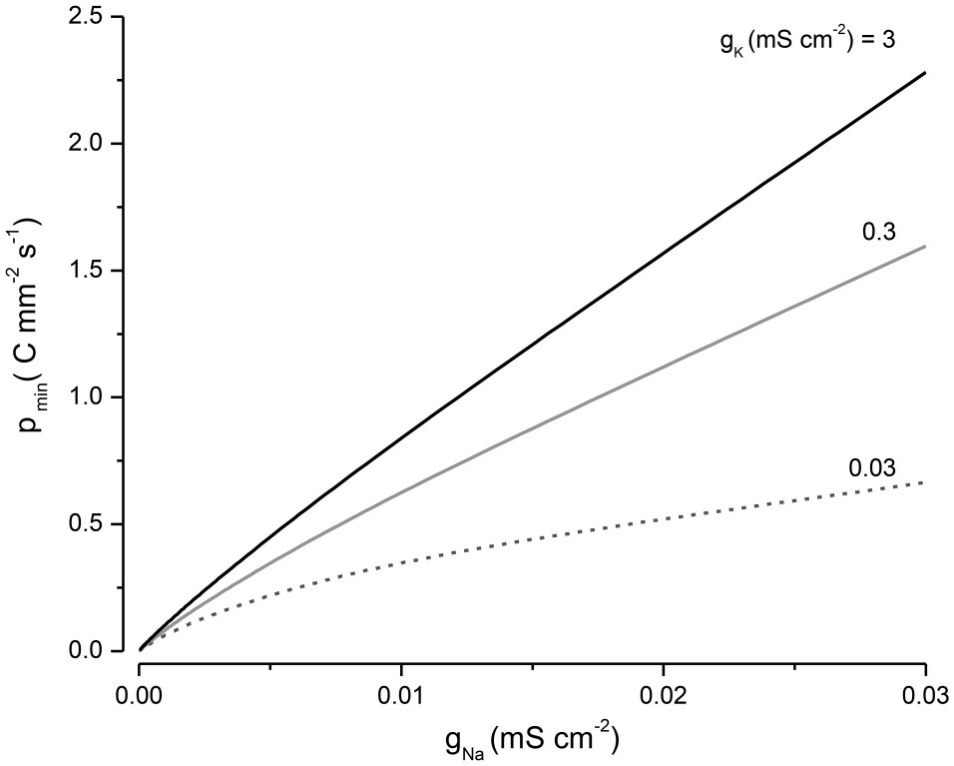
**The influence of ***g***_***Na***_ and ***g***_***K***_ on ***p***_***min***_**.

Equation (9) encapsulates how the energy required to maintain cell volume depends on the systems parameters. *p*_*min*_ can be minimized by keeping *g*_*Na*_ small but is less sensitive to the magnitude of *g*_*K*_ (Figure 6). It is worth noting for the case where *q* = 0 the volume declines to a minimum but does not increase as *p* is increased.

### An equivalent electrical circuit representation of the PLM

**Figure 7:**
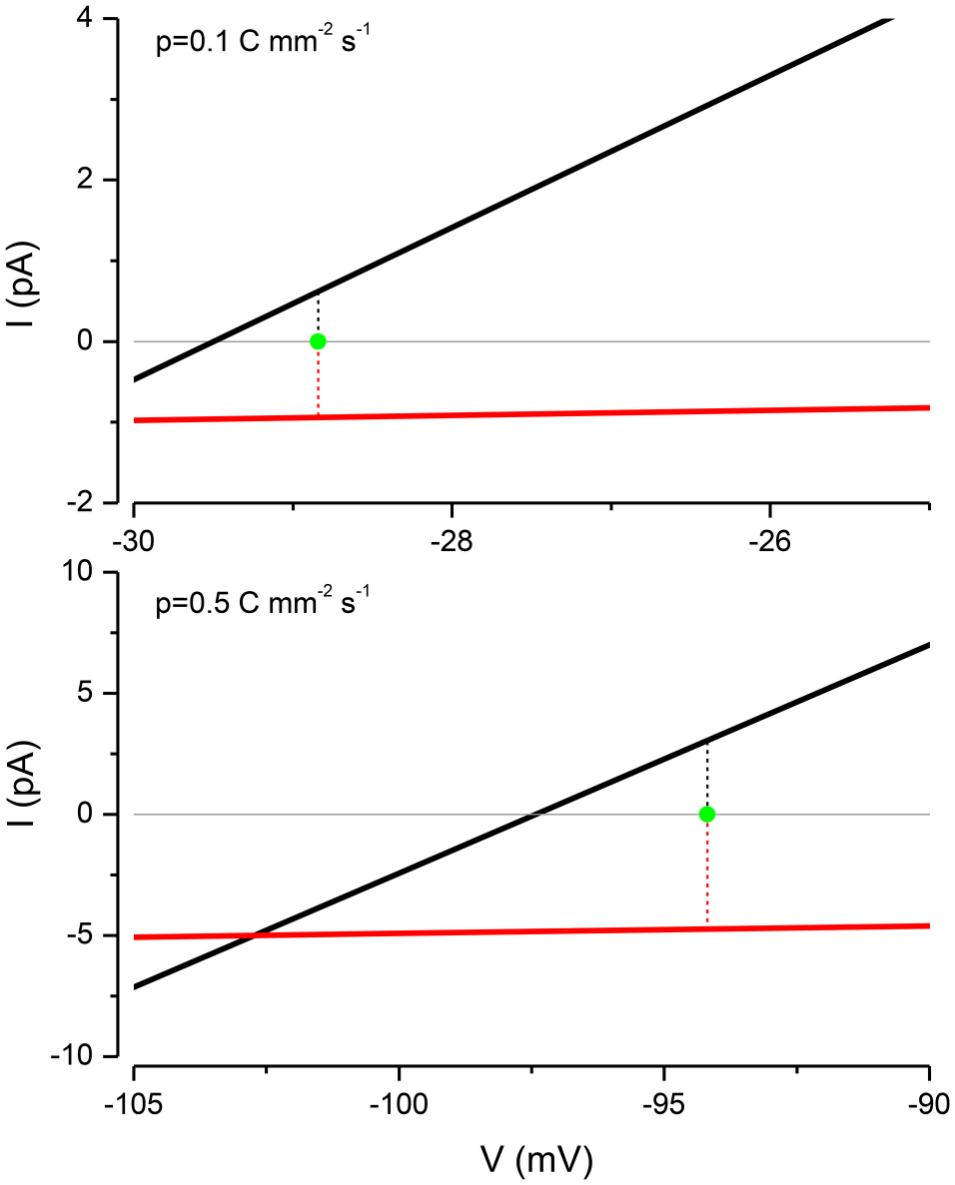
**The PLM balances the Na^**+**^ and K^**+**^ currents to stabilize the membrane potential**. For *p* = 0.1 C mm^−2^ s^−1^, *E*_*K*_ = −29.5 mV, *E*_*Na*_ = 1.155 mV and *V* = −28.84 mV and for *p* = 0.5 C mm^−2^ s^−1^, *E*_*K*_ = −97.43 mV, *E*_*Na*_ = 56.41 mV and *V* = −94.187 mV.

A standard method for predicting the electrical properties of cells is to build up an equivalent circuit model using capacitors, batteries and conductances; where the latter are often voltage gated (Kay, 2014). Because of ion accumulation and the variable volume, there is no simple way of devising such a circuit to represent the PLM. However a partial circuit representation can be developed, if I use the KSEs to estimate the ion distribution and steady state voltage at a given pump rate. A current-voltage (*IV*) relationship of the Na^+^ and K^+^ currents can then be used to show how current flow is apportioned. This is shown in Figure 7 for two different values of the pump current. The Nernst potentials of the K^+^ (i.e., $\frac{RT}{F}ln\left(\frac{K_{o}}{K}\right)$) and Na^+^ (i.e., $\frac{RT}{F}ln\left(\frac{Na_{o}}{Na}\right)$) conductances can be calculated from the their respective intra- and extracellular concentrations. Notice that at the resting potential both the passive Na^+^ and K^+^ currents are exactly balanced by an equal and opposite pump current, in the ratio 3:2. The system is stable because deviations from the resting potential will induce currents that restore it to rest.

### Pumps other than the NKA can stabilize cell volume

**Figure 8:**
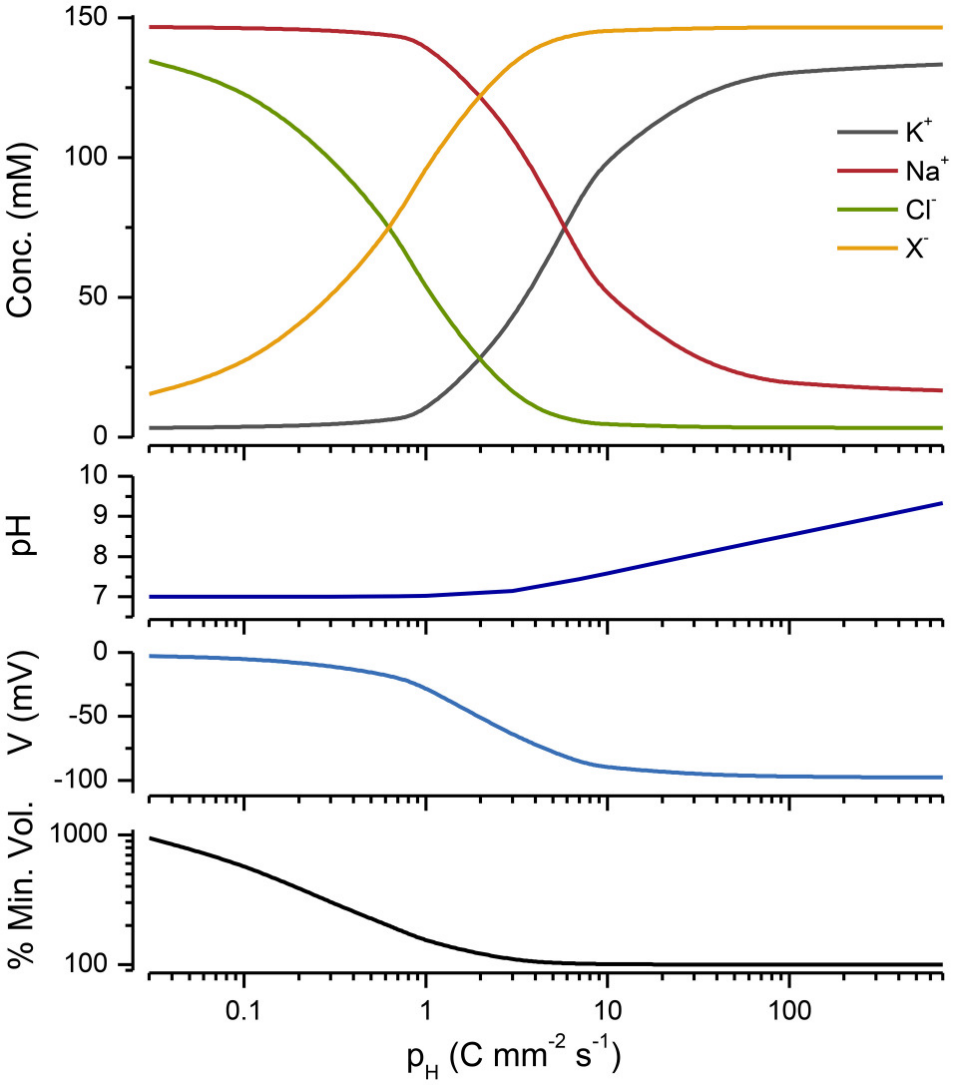
**A ***Cp*** plot of a PLM model incorporating a Na/H exchanger, a proton pump and conductance (see Appendix A7)**. The extracellular pH = 7, *p*_*Na*/*H*_ = 10^5^ and *g*_*H*_ = 0.007 mS cm^−2^ and *D*_*H*_ = 10^−7^M. The % of the minimum volume is plotted as function of *p*_*H*_ on the lowest panel.

The PLM is not exclusively dependent upon the operation of a NKA; there are other pumps that can substitute. All that is required is a mechanism for pumping Na^+^ out of the cell, together with passive Na^+^, K^+^, and Cl^−^ conductances, and water permeability. Mycoplasma, which are bacteria that do not have cell walls, provide a nice example for looking at alternative PLMs. Like other bacteria and archea they do not have a NKA, but they do have a proton pump (Krulwich et al., 2011) and a Na^+^/H^+^ transporter (Padan and Landau, 2016), where the proton gradient drives the outward flux of Na^+^. Figure 8 shows that the operation of a proton pump in conjunction with a Na^+^/H^+^ exchanger can stabilize a cell. Blocking the proton pump induces mycoplasma to lyse (Linker and Wilson, 1985).

## Discussion

Cell size regulation is a fundamental aspect of both single and multi-cellular organisms. Although the link between active and passive ion fluxes and cell size has been known for over 50 years, it seems not to have had much impact in cell biology. In as much as cell biologists, outside of neuroscience, typically do not seem to reach for electrophysiological explanations, in accounting for cellular phenomena.

From the components of the PLM it is difficult to discern how it stabilizes cell volume. The mechanism only become evident when one embodies it in a mathematical model and simulates it as a complete system. Although the essentials of the PLM have been around for a long time, its workings have not been widely spread in the form of textbook accounts, with some few exceptions (Stein, 1990; Weiss, 1996). Cell biology textbooks typically only offer verbal explanations that provide a rather superficial understanding of its mechanism of operation. It is perhaps worth noting that inattention to the importance of electrical current flow in cell biology proved a roadblock to the acceptance of a seminal breakthrough in cell biology, viz. Peter Mitchell's chemiosmotic hypothesis (Mitchell, 2004).

A central point to emerge from my analysis of the PLEs is that *x* determines the cell volume (see Equation 3). Therefore cells can potentially use *x* to regulate their volume. A plausible mechanism is for cells to continuously monitor their size and use a feedback mechanism to control their size. For example the concentration of an osmolyte like taurine could be increased or decreased depending on whether the size is below or above the size set point. There is evidence for active size sensing in yeast and a number of mechanisms have been proposed to account for it (Pan et al., 2014; Shahrezaei and Marguerat, 2015; Amodeo and Skotheim, 2016). A recent attempt to whittle down the genome of mycoplasma to an essential set of genes has revealed the presence of genes with unknown function (Hutchison et al., 2016). Might some of these be involved in cell size control?

I have not addressed the question of whether the PLM is the only mechanism that controls cell size. Some have claimed that viscoelastic properties of the cell membrane and cytoskeleton play a key role in cell volume regulation (Sachs and Sivaselvan, 2015). It seems to me that it is possible that both factors play a role in cell membrane stabilization.

Starting with Tosteson and Hoffman the PLEs have gone through a number of iterations (Mackey, 1975; Jakobsson, 1980; Lew et al., 1991; Kabakov, 1994; Hernandez and Cristina, 1998; Hoppensteadt and Peskin, 2002; Armstrong, 2003; Fraser and Huang, 2004; Takeuchi et al., 2006; Ataullakhanov et al., 2009; Yurinskaya et al., 2011). Mori (2012) has demonstrated that the PLEs have an asymptotically stable steady state so long as the pump current is not too large. While Vereninov et al. (2014) have shown how to estimate the parameters of a PLE from experimental data.

### The Donnan effect

The Donnan effect is frequently introduced in physiological textbooks, however the reader is often left hanging as to its precise implications for cell function. As I have shown, the effect of trapped molecules within the cell has a significant impact on the behavior of the cell; but it should be emphasized that a true (i.e., with no energy input) Donnan equilibrium cannot be attained, unless the membrane can sustain a very high transmembrane pressure. In the hypothetical case where Na^+^ is pumped actively while K^+^ and Cl^−^ distribute passively, these ions distribute in accord with the Donnan relationship (see above), the system is in a dynamic steady-state. Misunderstanding the Donnan effect, as has been pointed out by others (Voipio et al., 2014), can lead to erroneous ideas about the influence of impermeant ions on ion distributions.

It is worthwhile making a distinction between the PLM and the so-called Double-Donnan mechanism, because these two are sometimes conflated. The Double-Donnan mechanism (Leaf, 1959) proposes that the asymmetric ion distribution is achieved by the operation of a NKA together with a membrane that is permeable to K^+^, Cl^−^ and water but not to Na^+^. It is probably best to avoid this term since all membranes have a finite permeability to Na^+^. Moreover, if the sodium conductance is zero, the system of equations is unstable, with the Na^+^ concentration becoming negative, which is clearly impossible.

*x* is a heterogeneous collection of molecules, yet both it and *z* can be calculated. To do this one needs to have good estimates of the extracellular osmolarity and the intracellular concentrations of the predominant permeable anions and cations. One can then use Equation (1) to estimate *x* and Equation (2) *z*. The estimates are of course only as good as the intracellular concentration estimates, which as yet are difficult to obtain, absent sensitive and accurate monovalent ion sensors. Estimates of *z* have been made for muscle and some cell lines and ranges between −2 and −0.7 (Burton, 1983a; Model, 2014). Both *x* and *z* are unlikely to be fixed and will vary as impermeant molecules are metabolized or transported and as the pH changes. A precise accounting for the contributions to *z* has yet to be done. The contributions of nucleic acids and proteins will depend on how tightly counter ions bind (Raspaud et al., 2000). Some texts refer to *x* as the “anion gap,” which is sometimes incorrectly attributed to intracellular proteins.

### The role of leaks and the energetics of the PLM

If one is unaware of the role of the NKA in volume regulation, the presence of Na^+^, K^+^, and Cl^−^ leaks seems counterproductive as they impose an energetic load on the cell. It is only if one takes into account the central role of the NKA in volume regulation, that the function of the leak conductances become apparent as key components of the PLM. There are a large variety of leak channels (Ren, 2011; Feliciangeli et al., 2015) and it seems likely that they may have evolved to help stabilize cell volume. Moreover, *g*_*Na*_ has a profound effect on the energy utilization of a cell, with energy consumption in direct proportion to *g*_*Na*_ (Figure 6). Driving the NKA is one of the most energetically costly processes for animals, with an estimated 20% of a cell's energy consumption needed to keep it running (Rolfe and Brown, 1997; Milo and Phillips, 2015). This large energy investment is a clear indication of the importance of the PLM.

As I have shown, there is a minimum pump rate to achieve a stable volume. Operating above this rate does not change the volume much. As the pump rate declines below *p*_*min*_, the volume increases monotonically; however, even for very low pump rates, the volume is stable. This constitutes something of a problem for evaluating the PLM experimentally; even with very low pump rates the volume may be stable. In addition it is possible that other pumps may take over and sustain the volume. Moreover, as my simulations show (Figure 2), it may take a long time for ion gradients to dissipate after the application of a pump blocker like ouabain.

It is worth noting that the leak current is also an essential part of the mechanism that makes cells excitable. If one were to remove all leak currents, which is clearly not possible, from a cell with voltage gated Na^+^ and K^+^ channels, there would be no stable resting potential (Kay, 2014). Furthermore the PLM provides a simple explanation for the small changes in axon dimensions induced by action potential propagation (Cohen, 1973; Lee and Kim, 2010).

Equivalent circuit models are often used to simulate neurons, but most models do not, with some exceptions (Kager et al., 2002; Ostby et al., 2009; Ullah et al., 2015), incorporate water and ion fluxes, impermeant molecules and distensible membranes. Cellular swelling occurs after cerebral trauma, stroke, spreading depression and epilepsy (Rungta et al., 2015; Stokum et al., 2016). There is a pressing need for pharmacological interventions that limit swelling and prevent the damage resulting from cells being damaged while expanding into the closed volume of the cranium. Developing such agents requires a detailed understanding of all the factors that play a role in cell volume regulation and the theory that undergirds this mechanism. The operation of the PLM in excitable cells like neurons, with more vigorous passive ion fluxes than in other cells, imposes a more severe energetic demand on neurons and muscle. This by itself may explain why neurons are so sensitive to oxygen deprivation.

The maintenance and restoration of Na^+^ and K^+^ gradients is the most conspicuous function of the NKA, yet its role in volume regulation is seldom recognized. Stein (1995) has argued that the evolution of the NKA and the PLM is a defining characteristic of animal cells. It endows them with an osmoregulatory mechanism that does not require rigid cells and it gives them a negative resting potential, which turned out to be very convenient for evolving excitable cells (Jakobsson, 1980; Armstrong, 2015). The PLM also liberates cells from the straitjacket of cell walls allowing the evolution of contractile mechanisms.

## Author contributions

AK initiated the project, analyzed the equations, performed simulations and wrote the paper.

### Conflict of interest statement

The author declares that the research was conducted in the absence of any commercial or financial relationships that could be construed as a potential conflict of interest.

## A. Appendix

### A.1. The Keener-Sneyd Solutions

Here I will present the equations that were derived by Keener and Sneyd (2009) with some new extensions that I have deduced (viz. for the case where *z* = −1, the instability of the system when *x* = 0 and the effect of knocking out Na^+^ or K^+^ transport). The NKA is assumed to operate at a constant rate *p*, with *n* and *q* the number of Na^+^ and K^+^ ions transported per pump cycle.

At steady state each of the current flows is zero and the system of Equations (1, 2, 4–6) can be solved exactly for the five unknowns *Na, K, Cl, w*, and *V*:

I follow Keener and Sneyd in defining the following non-dimensional variables:

(A1) $$\begin{array}{l} \nu =\frac{FV}{RT},\\ P=\frac{pF}{RTg_{Na}},\\ \mu =\frac{w}{x}Cl_{o} \end{array}$$

I define:

(A2) $$\begin{array}{l} \alpha =\frac{Na_{o}e^{-nP}+K_{o}e^{qP\gamma }}{Na_{o}+K_{o}} \end{array}$$

where $\gamma {=}^{g_{na}}{/}_{g_{K}}$

(A3) $$\begin{array}{l} y=e^{-\nu } \end{array}$$

Keener and Sneyd demonstrated that for $n\frac{Na_{o}}{g_{Na}}>q\frac{K_{o}}{g_{K}}$ there is a range of *p* for which the system has a finite positive cell volume, with the following solutions:

(A4) $$\begin{array}{l} \mu =\frac{1+\sqrt{1-\left(1-\alpha \right)\left(1-z^{2}\right)}}{2\left(1-\alpha \right)} \end{array}$$

(A5) $$\begin{array}{l} y=\frac{-z+\sqrt{z^{2}+\;4\alpha \mu^{2}}}{2\alpha \mu } \end{array}$$

(A6) $$\begin{array}{l} Na=Na_{o}ye^{-nP} \end{array}$$

(A7) $$\begin{array}{l} K=K_{o}ye^{qP\gamma } \end{array}$$

(A8) $$\begin{array}{l} Cl=Cl_{o}/y \end{array}$$

*For the case where* *z* = −1

From Equations (1) and (2):

(A9) $$\begin{array}{l} K+Na=Cl+X=0.5\;\Pi_{o} \end{array}$$

Then from Equations (A6) and (A7)

(A10) $$\begin{array}{l} K_{o}\text{y}e^{qP\gamma }+Na_{o}\text{y}e^{-nP}=0.5\;\Pi_{o} \end{array}$$

solving for *y*

(A11) $$\begin{array}{l} y=\frac{0.5\Pi_{o}}{K_{o}e^{qP\gamma }+Na_{o}e^{-nP}}=1/\alpha \end{array}$$

from (A4) and (A11)

(A12) $$\begin{array}{l} \mu =\frac{1}{1-\alpha }=\frac{y}{y-1} \end{array}$$

### A.2. A more realistic mathematical representation of the NKA

In the Keener-Sneyd equations the NKA is represented by as constant term (Equations 4 and 5). To provide a more realistic representation of the NKA I incorporated its dependence on K^+^ and Na^+^ concentrations (Truskey et al., 2009). The Na^+^ flux is:

(A13) $$\begin{array}{l} J_{NKA,Na}=-p\prime {\left(\frac{Na}{D_{Na}+Na}\right)}^{3}{\left(\frac{K_{o}}{D_{K}+K_{o}}\right)}^{2} \end{array}$$

The K^+^ flux is:

(A14) $$\begin{array}{l} J_{NKA,K}=-\frac{2}{3}J_{NKA,Na} \end{array}$$

where: *p*′ is the maximum Na^+^ efflux at steady state, *D*_*Na*_ and *D*_*K*_ are the apparent dissociation constants for Na^+^ and K^+^. Figure A1 shows that the form of the *C*−*p* curve is maintained even with this nonlinear pump mechanism.

### A.3. No impermeant molecules (*x* = 0)

Although this is clearly an impossible situation it is worth considering because it can provide insight into the action of *x* in the PLM.

From Equations (1) and (2).

(A15) $$\begin{array}{l} Na+K=Cl=Cl_{o}=\Pi_{o} \end{array}$$

When the NKA is not operating from Equation (6), *V* = 0, *Na* = *Na*_*o*_, *K* = *K*_*o*_ and the volume is stable. When the pump is on from Equations (4) and (5) one can calculate the distribution of Na^+^ and K^+^ as a function of pump rate and from that the membrane potential, which becomes more negative as *p* increases. However *Cl* = *Cl*_*o*_ and there is no way around that. The only thing that can give is the volume, so as one turns on the pump the volume decreases and if it is kept on, the volume will go to zero.

### A.4. The braided structure of the solution to the PLEs with *z* = −1

For the case where *z* = −1 from (1) and (2) as *p* is increased the following crossovers occur in sequence (see Figure 5 second panel from the top) and it is possible to derive analytical expressions for *p* at all the intersection points for the case where there is no active K^+^ transport (i.e., *q* = 0):

For *Na* = *Cl* and *K* = *X*:

(A16) $$\begin{array}{l} p=-\frac{g_{Na}RT}{nF}ln\frac{K_{o}\left(\Pi_{o}-{\left(\Pi_{o}^{2}-16K_{o}Cl_{o}\right)}^{½}\right)}{Na_{o}\left(\Pi_{o}+{\left(\Pi_{o}^{2}-16K_{o}Cl_{o}\right)}^{½}\right)} \end{array}$$

for *Cl* = *X* = 0.5Π_*o*_:

(A17) $$\begin{array}{l} p=-\frac{g_{Na}RT}{nF}ln\left(\frac{\frac{\Pi_{o}^{2}}{8Cl_{o}}-K_{o}}{Na_{o}}\right) \end{array}$$

for *Na* = *X* and *K* = *Cl*: For *K* = *Cl*

(A18) $$\begin{array}{l} p=-\frac{g_{Na}RT}{nF}ln\left(\frac{0.5\Pi_{o}{\left(\frac{K_{o}}{Cl_{o}}\right)}^{½}-K_{o}}{Na_{o}}\right) \end{array}$$

for *Na* = *K* = 0.5Π_*o*_:

(A19) $$\begin{array}{l} p=-\frac{g_{Na}RT}{nF}ln\left(\frac{K_{o}}{Na_{o}}\right) \end{array}$$

for *Na* = *Cl* and *K* = *X*:

(A20) $$\begin{array}{l} p=-\frac{g_{Na}RT}{nF}ln\frac{K_{o}\left(\Pi_{o}+{\left(\Pi_{o}^{2}-16K_{o}Cl_{o}\right)}^{½}\right)}{Na_{o}\left(\Pi_{o}-{\left(\Pi_{o}^{2}-16K_{o}Cl_{o}\right)}^{½}\right)} \end{array}$$

### A.6. Mathematical knockout of the components of the PLE

*No Na*^+^ *pumping* [*z* = −1, *n* = 0]

Equation (A11) becomes:

(A21) $$\begin{array}{l} y=\frac{0.5\Pi_{o}}{K_{o}e^{qP\gamma }+Na_{o}} \end{array}$$

*y* ≤ 0 and from Equation (A8) *Cl* ≥ *Cl*_*o*_, which with Equations (1) and (2) implies that *X* ≤ 0 which means that *x* cannot be accommodated and hence the system is unstable.

*No K*^+^ *pumping* [*z* = −1, *q* = 0]

From Equations (A7) and (A8)

(A22) ClClo=KoK

Which is the Donnan ratio.

From Equation (A11)

(A23) $$\begin{array}{l} y=\frac{0.5\Pi_{o}}{K_{o}+Na_{o}e^{-nP}} \end{array}$$

Substituting this into Equation (A6) gives:

(A24) $$\begin{array}{l} Na=\;\frac{0.5\;Na_{o}\Pi_{o}}{K_{o}e^{nP}+Na_{o}} \end{array}$$

and substituting Equation (A23) into Equation (A7) gives:

(A25) $$\begin{array}{l} K=\;\frac{0.5\;K_{o}\Pi_{o}}{K_{o}+Na_{o}e^{-nP}} \end{array}$$

### A.7. Mycoplasma model

To model mycoplasma I added an outward directed proton pump, a Na^+^/H^+^ exchanger and a proton leak in the PLM model.

To model the Na^+^/H^+^ exchanger I assume that it can be described by the following chemical equilibrium:

(A26) $$\begin{array}{l} H_{o}+Na⇆H+Na_{o} \end{array}$$

Where *H* and *H*_0_ are the inside and outside proton concentrations respectively.

The proton flux through the exchanger is then:

(A27) $$\begin{array}{l} J_{NaH,H}=p_{NaH}\left(Na_{o}H-NaH_{o}\right) \end{array}$$

Where *p*_*NaH*_ is the permeability of the exchanger. Similarly the Na^+^ flux though the exchanger is:

(A28) $$\begin{array}{l} J_{NaH,Na}=-p_{NaH}\left(Na_{o}H-NaH_{o}\right) \end{array}$$

The outward proton pump is represented by the following equation:

(A29) $$\begin{array}{l} J_{H}=p_{H}\frac{H}{H+D_{H}} \end{array}$$

where *p*_*H*_ is the strength of the pump and *D*_*H*_ is the dissociation constant for protons.

In this model the rate of change of intracellular proton concentration is then:

(A30) $$\begin{array}{l} \frac{dH}{dt}=-\frac{A}{wF}\left({\text{g}}_{H}\left[V-\frac{RT}{F}ln\left(\frac{H_{o}}{H}\right)\right]+J_{H}+J_{NaH,H}\right) \end{array}$$

Where g_*H*_ is the proton conductance.

The rate of change of intracellular Na^+^ is then:

(A31) $$\begin{array}{l} \frac{dNa}{dt}=-\frac{A}{wF}\left({\text{g}}_{Na}\left[V-\frac{RT}{F}ln\left(\frac{Na_{o}}{Na}\right)\right]+J_{NaH,H}\right) \end{array}$$

The proton and hydroxide (calculated assuming that the ionic product of water is 10^−14^ M^2^) ion concentrations are included in Equations (1), (2), and (8).

## Abbreviations

CD: charge-difference
KSS: Keener-Sneyd solutions
NKA: Na^+^/K^+^ ATPase
PLE: pump-leak equations
PLM: pump-leak mechanism.
